# Blood viscosity as a forgotten factor and its effect on pulmonary flow

**DOI:** 10.1186/2213-0802-1-3

**Published:** 2013-02-22

**Authors:** Gulfidan Cakmak, Fatma Ates Alkan, Kazim Korkmaz, Zuhal Aydan Saglam, Denizhan Karis, Mustafa Yenigun, Meltem Ercan

**Affiliations:** 1 Department of Chest Disease, Haseki Training and Research Hospital, Millet St Fatih, Istanbul, 34096 Turkey; 2 Department of Biophysics, Istanbul University Cerrahpasa Medical Faculty, Fatih, Istanbul, 34098 Turkey; 3 Department of Cardiology Disease, Haseki Training and Research Hospital, Millet St Fatih, Istanbul, 34096 Turkey; 4 Department of Family Practitioners, Haseki Training and Research Hospital, Millet St Fatih, Istanbul, 34096 Turkey; 5 Department of Internal Disease, Haseki Training and Research Hospital, Millet St Fatih, Istanbul, 34096 Turkey

**Keywords:** Chronic Obstructive Pulmonary Disease, Pulmonary Hypertension, Right Ventricle, Force Vital Capacity, Thyroid Stimulate Hormone

## Abstract

**Background:**

The effect of smoking on blood viscosity is widely known. There are, however, few studies on the effect of blood viscosity on pulmonary circulation.

**Methods:**

We aimed to observe the relationship between blood viscosity and pulmonary circulation among smokers and non-smokers. The study comprised 114 subjects in three groups: group 1, ex-smokers; group 2, smoked at least 10 packs/year and still smoking; group 3, never smoked. Blood viscosity (BV), pulmonary blood flow (PBF), and right ventricular systolic pressure (RVSP) were measured in all subjects.

**Results:**

PBF was significantly lower in group 1 compared with group 3 (p < 0.05). BV in group 1 was significantly higher than group 3 (p < 0.05) while BV in group 2 was significantly higher than group 3 (p < 0.05). PBF in group 2 was significantly lower than group 3 (p = 0.01).

**Conclusions:**

We believe that BV is a significant and forgotten factor that plays an important role in pulmonary and cardiovascular diseases. BV may affect PF even during the course of smoking, and before the clinical onset of chronic obstructive pulmonary disease (COPD). Therefore, individuals at risk of pulmonary hypertension could be detected earlier with a simple blood test.

## Background

Cardiovascular and pulmonary diseases are a significant cause of mortality worldwide [1–4]. Despite efforts to control their causes, these diseases are still common and often fatal. These data suggest that forgotten or neglected etiological factors may be responsible. We believe that blood viscosity (BV) may be an important factor. To supply adequate oxygen and nutrients to the tissue and organs, the blood flow rate needs to be of a normal pattern and quantity within the vessels [5–9]. BV is an important factor contributing to normal vessel flow and can be affected by multiple factors, including hematocrit and smoking. BV has been studied in COPD, pulmonary hypertension, anemia, and other blood diseases [10–13]. We aimed to evaluate the relationship between BV, pulmonary flow rate (PFR), pulmonary artery pressure (PAP) and right ventricular systolic pressure (RVSP) in subjects with normal spirometric values, no hypoxemia or COPD, and no disease or drug usage affecting BV. Thus, we aimed to determine whether BV could be used to screen healthy individuals, and to follow-up those individuals with a risk of pulmonary hypertension before disease onset.

## Methods

### Study design

The study was initiated following ethics committee approval from the Istanbul University Cerrahpasa Medical Faculty. One hundred and fourteen subjects between 35–65 years attending the Clinic of Chest Diseseas as outpatients with any reason and their healthy volunteering relatives or friends between January and June 2011 were enrolled in the study. Written consent was obtained from every individual. Attendants who had any systemic disorder and/or who had body-mass index (BMI) greater than 25kg/m^2^ and/or who were consuming any drugs which might be effecting blood viscosity were excluded. People who had abnormal biochemical values of fibrinogen, cholesterol, creatinine, thyroid hormones, and whole blood count were excluded as well. Blood samples were taken for biochemical parameters, whole blood counts, and BV. Spirometric values were measured, and PFR, PAP and RVSP were determined by means of echocardiography. Although catheterization is the best technique for measuring PAP, we preferred echocardiography as a non-invasive method in healthy individuals. One hundred and fourteen subjects with no history of any disease and/or any medications that would affect BV, and with normal biochemical parameters, whole blood count, and spirometry (without COPD), were enrolled in the study.

We divided the subjects into three groups: group1, ex-smokers (n = 40); group 2, current smokers with at least 10 pack years exposure (n = 34); group 3: never-smokers (n = 40).

### Methods

Tri-iodotyronine (fT_3_), thyroxine (fT_4_) and thyroid stimulating hormone (TSH) were assessed with the Advia Centaur XP immunoassay device from Siemens using chemiluminescence, as per the manufacturer’s instructions. fT_3_ and fT_4_ were expressed as ng/dL, and TSH was expressed as mIU/L. Other biochemical parameters were studied using spectrophotometry with the Advia 2400 device from Siemens Company. Fibrinogen was assessed using the Claus method with the MDA 180 device (Trinity Biotech Company) and expressed as mg/dL. The hemogram was studied using electro-impedance with a Coulter LH 780 device (Beckman Coulter Company). Hemoglobin was expressed as g/dL, hematocrit as %, and leucocyte count was expressed as 10^3^/mL.

BV was measured with Brookfield LVDVIII rotational viscosimetry at a constant 37°C and at three different shear rates (23, 115.2, 230 sn^-1^). Results were expressed as mPas. To eliminate the effect of hematocrit, which is accepted as one of the most important factors affecting BV, it was adjusted to a hematocrit of 45% using the Quemada equation [10]. These adjusted BV values were termed ‘corrected BV’, and were reported at shear rates of 23, 115.2 and 230 l/sec. Spirometric measurements were performed using the ZAN 100 USB flow handy device. Vital capacity (VC), forced vital capacity (FVC), maximum forced expiratory volume in one second (FEV_1_), mean forced expiratory flow during mid-half of the FVC (FEF_25-75_), and FEV_1_/FVC were measured. PBF and RVSP were measured by means of the E Sante My Lab 60 device using a 2.5 MHz frequency transducer. Systolic mean blood flow velocity at the pulmonary valve was measured as m/s with pulse wave Doppler from the parasternal short axis. RVSP was measured with continuous wave (cv) Doppler from the apical four chambers, and tricuspid insufficiency was demonstrated by detecting jet-stream. RVSP was calculated by adding right atrium pressure to the right ventricle (RV)-right atrium (RA) gradient by means of the Bernoulli principle, and expressed in mmHg.

### Analysis

For statistical analysis, NCSS (Number Cruncher Statistical System) 2007&PASS 2008 Statistical Software (Utah, USA) was used. Besides descriptive statistical methods (mean, standard deviation = SD), one-way Anova was used for intergroup comparisons of quantitative data with normal distribution. For analysis of variance Tukey HSD was used, and for relationships between parameters the Pearson’s correlation coefficient was used. Data were presented as mean±SD. Results were noted as significant if p < 0.05.

## Results

Demographic details and basic biochemical characteristics of the different groups are shown in Table 1. There were no significant differences between the groups in terms of gender, age, body mass index (BMI), and PAP (group 1, 20F/20 M, age was 51±8 yrs; group 2, 15F/19 M, age was 44±7 yrs; and group 3, 23F/17 M, age was 47±7 yrs). There were also no differences in the RVSP between the groups.

**Table 1:** **Demographic and biochemical parameter of groups**

	Group1(n=40)	Group2(n=34)	Group3(n=40)
Age(year)	51±8	44±7	47±7
Sex(F/M0	20/20	15/19	23/17
BMI(kg/m^2^)	27.±4.3	27.9±5.4	58.2±4.8
SBP(mmHg)	96±16	95±15	92±16
DBP(mmHg)	70±5	75±7	73±6
Hemoglobin(g/dl)	14.20±1.40	14.80±1.52	12.94±1.48
Hemotocrit(%)	42.58±3.99	43.58±9.47	39.07±5.98^a*,b**^
FT_3_(ng/dl)	3.02±0.50	3.21±0.47	3.03±0.45
FT_4_(ng/dl)	1,18±0.21	1.17±0.15	1.14±0.13
TSH(mIU/L)	24.23±7.40	1.77±0.94^a**^	1.75±0.83^a**^
ALT(U/L)	24.23±7.40	23.00±6.68	17.90±5.58^a**,b***^
AST(U/L)	26.80±7.91	21.91±7.63^a*^	19.39±6.53^a***^
Creatinin(mg/dl)	0.90±1.14	4.64±1.06	4.33±1.40
Uric acid(mg/dl)	4.90±1.14	4.64±1.06	4.33±1.40
Cholesterol(mg/dl)	178.71±41.05	179.55±32.38	189.90±30.86
Triglyceride(mg/dl)	105.85±40.64	122.55±59.31	114.74±64.54
LDL(mg/dl)	112.35±36.51	111.93±29±86	119.11±26.21
HDL(mg/dl)	44.19±9.15	43.11±15.14	47.76±10.10^b*^
Glucose(mg/dl)	94.06±9.55	91.87±7.70	95.78±8.13
Fibrinogen(mg/dl)	405.90±111.60	344.55±78.29	380.86±81.84^b*^
Leukocyte(10^3^/ml)	7.39±1.92	7.50±1.60	6.80±1.42
Pulmonary flow rate	1.05±0.25	1.00±0.16	1.21±0,34^a**.b***^
Pulmonary pressure (mmHg)	10±2.2.5	12±2.35	9±1.98

SBP; Systolic bloodpressure, DBP; Diastolic blood pressure, Data are mean±SD, ^a^ Statistical comparison with Group 1, ^b^ Statistical comparison Group 2 with Group 3, * p<0,05, **p<0,01, ***p< 0,001.

Pulmonary blood flow was 1.05±0.25, 1.00±0.16, and 1.21±0.34 in the three groups respectively. There was no statistically significant difference in PFR between groups 1 and 2, but PFR in group 3 was significantly higher than groups 1 and 2 (p < 0.01 and p<0.001, respectively) (Table 1).

When the hematocrit values were examined, no statistically significant difference was found between group 1 (42.58±3.99%) and group 2 (43.58±9.47%). However, the hematocrit in group 3 (3.07±5.98%) was significantly lower than the other two groups (Table 2).

**Table 2:** **The values of blood viscosity and blood viscosity standardized hct at three different shear rate of groups**

	Group 1(n=40)	Group 2(n=34)	Group 3(n=40)
Standardized			
Hematocrit(%45)	9.28±1.95	9.49±1.69	8.85±1.35 ^a*,b**^
BV (m Pas)	6.26±1.04	6.33±0.91	5.87±1.27
BV (mPas)	4.30±0.51	4.41±0.47	4.04±0.31 ^b**,a***^

Data are mean±SD, BV ; Blood viscosity at shear rate 23 1/sec, BV; ; Blood viscosity at shear rate 115,2 1/sec, BV; Blood viscosity at shear rate 230 1/sec, ^a^ Statistical comparison with Group 1, ^b^ Statistical comparison Group 2 with Group 3, * p<0,05, **p<0,01, ***p< 0,001.

When BV was measured after the hematocrit was standardized at 45%, the viscosities of group 2 (6.18±1.12 mPas) and group 3 (6.99±0.97 mPas), which represented middle vessels, were significantly different from that when the hematocrit was not standardized (p < 0.001). There was no statistical significant difference observed in terms of BV between the groups (p > 0.05) (Table 2).

When the spirometric values of all the groups were examined, FVC (%) of group 1 versus 3, and group 2 versus 3 showed a statistically significantly higher (p < 0.05 and p < 0.001, respectively). FEV_1_ (%) was only significantly different when group 2 and 3 were compared (61.76±20.92 vs 89.5±24.71, p < 0.05) (Table 3).

**Table 3:** **Spirometric values of groups**

	Group 1(n=40)	Group 2(n=34)	Group 3(n=40)
FVC	3171.6±963.12	2963.25±1031.17	3248.33±662.30
FVC(%)	83.83±13.55	81.23±20.35	94.42±23.43a*,b**
FEV_1_	2430.00±708.57	2166.75±818.10	2509.41±796.63
FEV_1_ (%)	76.76±19.60	71.70±20.92	89.50±24.71b*
FEV_1_/FVC	76.41±8.53	73.05±13.95	79.55±7.54

Data are mean±SD, FVC; Forced Vital Capacity, FEV1; Forced Expiratory Vital Capacity, FVC; Forced Percentual Vital Capacity, FEV_1_, Forced Percentual Expiratory Volume, FEV_1_/FVC; Tiffeneau Index, ^a^ Statistical comparison with Group 1, ^b^ Statistical comparison Group 2 with Group 3, * p<0,05, **p<0,01.

When the hematocrit was standardized to 45% the BV was found to correlate with age, smoking, urea, cholesterol, and white blood cell count in group 1, and with age, smoking, and urea in group 2. BV only correlated with urea in group 3.

## Discussion

Blood can carry the necessary nutrients to tissues and organs as long as it continues its normal flow pattern in the circulatory system. This normal flow is particularly important in the pulmonary vascular bed in order that gas exchange can be performed optimally [14–17]. Vessel constitution and the fluidity of blood need to be normal for normal blood flow [18, 19]. BV is one of the major determinants of blood flow and can behave differently in vessels with different diameters. BV is primarily affected by hematocrit in medium and large sized vessels, while it is affected by red blood cell deformability in the capillaries [1–9].

High BV causes slowing of blood flow [9, 10, 12, 18]. In addition to its hematological implications, hematocrit is functionally important since it is the major determinant of BV [9, 11, 20–29]. Our results suggest a significant relationship between blood viscosity, hematocrit, smoking, age, urea, and spirometric parameters. The effect of smoking on BV has been reviewed in many studies [1, 7, 8, 13–16]. The mechanism of action of smoking involves airway obstruction, vascular endothelial dysfunction, an increase in BV and a subsequent decrease in pulmonary blood flow [1, 14, 15, 18–24]. A study by Wannamethee et al. demonstrated that BV is increased in cigarette smokers. Thus, smoking is suggested to be an important factor in BV, which is one of the leading hemostatic factors [25].

Consistent with other studies, hematocrit levels were found to affect BV, so to exclude this affect, hematocrit values were fixed to 45% using a standard formula, and an adjusted BV was calculated. Although the hematocrit effect was ruled out, the blood viscosities of both current and former smokers were still higher than the non-smoking group [8]. This finding suggests that BV may be emphasized as a risk factor [1, 16, 26, 27, 30–32]. Our results are consistent with previous relevant studies. Increased BV and decreased pulmonary blood flow play an important role in the progression of pulmonary hypertension (PH). PH causes cor pulmonale in chronic obstructive pulmonary disease (COPD) patients and has prognostic relevance [13, 16, 20]. Fedde et al. [31] demonstrated the relationship between BV, pulmonary blood flow, and pulmonary hypertension. Furthermore, Lenz et al. showed the association between hematocrit and pulmonary blood flow. Following hemodilution, pulmonary blood flow and exercise capacity improve [33]. Although there was no significant difference in RVSP between groups, we found blood flow decreased in smokers compared with non-smokers. According to the Poiseuille law [34], BV has a direct relationship with pressure gradient and vessel diameter, and an inverse relationship with blood flow and vessel length. Thus, pressure gradient and blood flow are directly proportional. Blood flow is linearly proportional with pressure gradient and inversely proportional with the vessel resistance. Blood flow is determined by the decrease of pressure from the arteries to the veins. The resistance is determined by vascular barrier and BV. An increase in BV causes an increase in vascular resistance, thus causing a decrease in blood flow. In light of the foregoing biophysical equations, we suggest that an increase in BV may cause a decrease in pulmonary blood flow, and as a result, an increase in pulmonary artery pressure. This finding may be relevant to the stage prior to the development of PH.

## Conclusion

In summary, smoking causes an increase in BV, which slows PBF. A decrease in PBF without an increase in RVSP may be an indicator of an early onset of PH. The results of our study in relation to individuals developing PH provide valuable data.

Previous studies have shown that BV is increased in COPD and hypoxemic patients but our study is unique and important, as the increase in BV could be detected in smokers before the onset of clinical disease. Thus, we believe that assessing the risk of PH in its very early stages may be possible. We wish to emphasize the importance of BV as a simple parameter for risk assessment in the early stages of PH, and cor pulmonale with respect to COPD associated mortality [35, 36].

This study is limited by its small patient number. Other parameters that could affect blood viscosity and hemodynamics are being studied with an increased number of patients.
